# A sudden-melting event during water freezing inside a copper well†

**DOI:** 10.1039/c8ra06601a

**Published:** 2018-10-15

**Authors:** WenQiang Xu

**Affiliations:** Department of Physics, The Chinese University of Hong Kong Hong Kong SAR People’s Republic of China newpo016@hotmail.com

## Abstract

We studied the freezing of super-cooled water inside a millimeter-sized copper well by confocal microscopy. During freezing, we surprisingly observed a novel melting scenario, which we call a ‘sudden-melting event’: the ice directly above the bottom substrate suddenly melts in the late stage of the freezing process, while the system is continuously being cooled. After this event, an empty gap around 10 μm to 20 μm between the substrate and the bulk ice is formed. Because this gap occupies the majority of the area of the bottom substrate, the adhesion between the bulk ice and the substrate is greatly reduced: the adhesion force decreases by more than 50% compared with the flat-substrate situation. We further discovered that air dissolved in water plays a crucial role in this melting event: the air excluded by water freezing produces inter-connecting channels in the bulk ice, which transport the warm water produced by latent heat to the substrate which causes the sudden melting event. Because this event makes the contact between ice and substrate very poor, and greatly reduces ice adhesion, our observation may lead to a promising anti-icing method on solid substrates. Compared to the prevalent super-hydrophobic surface technique, our approach only requires millimeter-sized wells instead of complex microscopic textures. Therefore, it is much easier and cheaper to produce, as well as much more robust for large-scale practical applications.

## Abstract 2

We observed a novel melting scenario by confocal microscopy, which we call a ‘sudden-melting event’, during the freezing of super-cooled water inside a millimeter-sized copper well.

## Graphical abstract

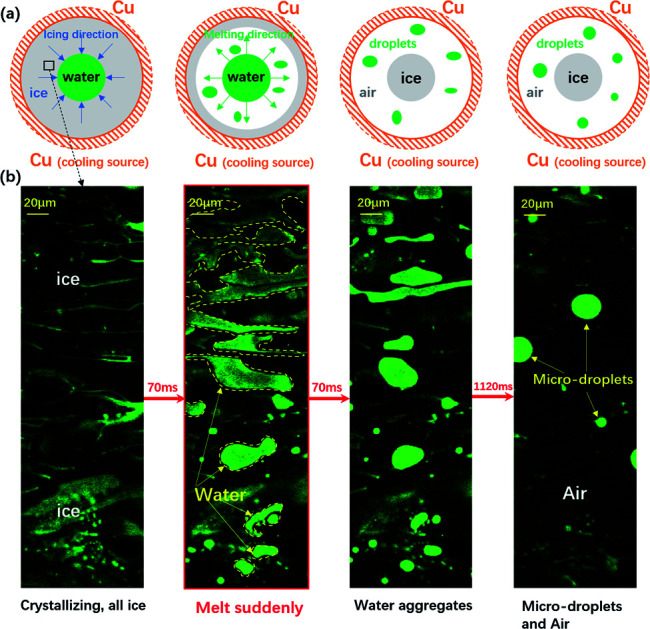

## 1 Introduction

The formation and subsequent accumulation of ice on surfaces affects the operation of numerous components of modern infrastructure, including ships, air-craft, offshore oil platforms, dams, wind turbines, power transmission lines, and telecommunications equipment, and introduces lots of damage and loss to our everyday life.^1–3^ Therefore, development of robust and easy anti-icing methods is always extremely important, which has attracted lots of attention from various researchers due to great significance in our daily life and in industry. To avoid icing, people initially tried to use well-suited materials from nature, like animal fur and natural fibres.^4,5^ These strategies were somewhat useful but not efficient. In the past several decades, with the help of advances in the understanding of liquid–solid interactions,^6–8^ to acquire ice-phobicity attention has been drawn to super-hydrophobic surfaces with fabrication of nanometer-sized or micrometer-sized textures,^9–15^ spraying hydrophobic organics (like proteins),^16–18^ adding various hydrophobic coatings,^19–22^ or even ferrofluids (combined with a magnetic field).^23^ These strategies show their efficiency in anti-icing under experimental conditions, but progress has been hindered by the relatively high cost to produce the textures at the nanometer and micrometer scales, the difficulties of maintaining such microscopic textures in a natural environment, and the Wenzel to Cassie transition of the surfaces,^24–29^ as well as mass production and large-scale applications. Instead of fabricating super-hydrophobic textures, here we report a novel method to directly decrease the contact area between ice and the substrate, from the aspect of surface profile design (a millimeter-sized copper well with a glass substrate attached) and thermodynamics (ice self-melting), and achieve a low-cost and highly-robust anti-icing function (more than a 50% ice adhesion force deduction). Our observations may potentially find broad applications in the anti-icing industry.

## 2 Methods

**Fig. 1:**
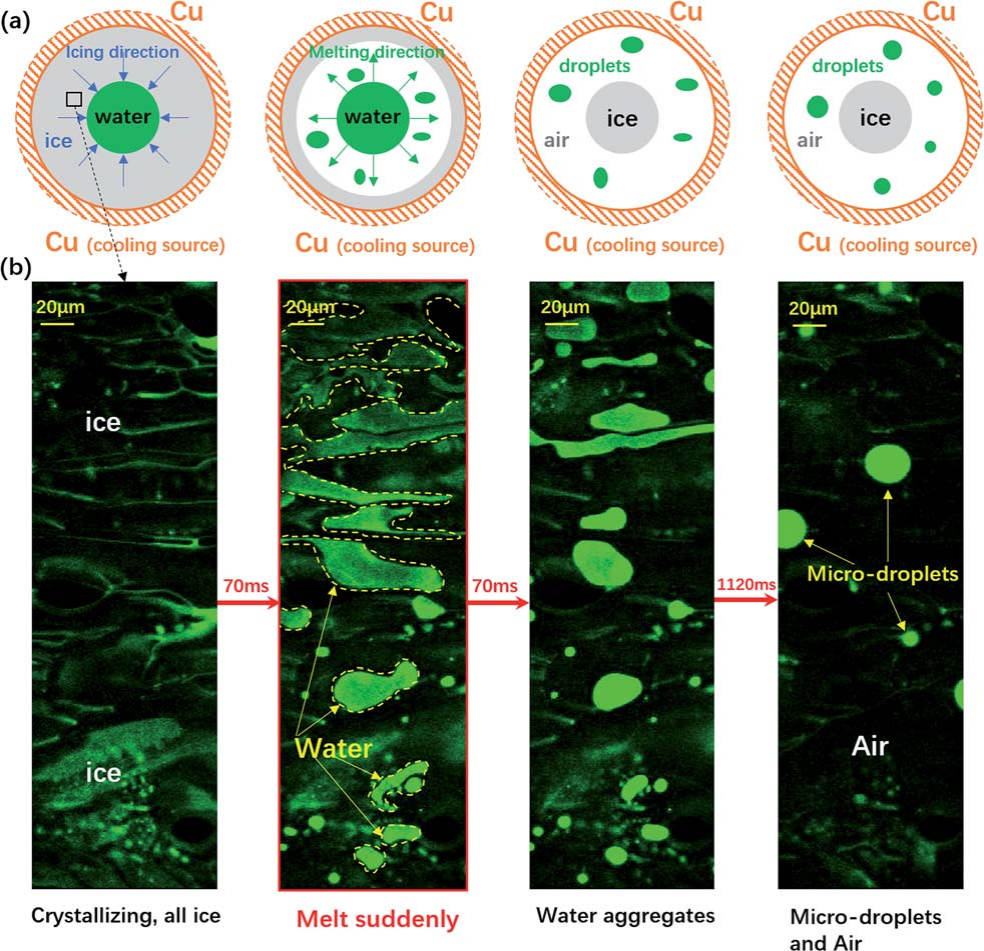
The sudden melting event captured by confocal microscopy. The focal plane is right above the water–substrate interface. (a) The overall schematic illustration of the sudden melting event at the bottom substrate. The setup was a copper well with a diameter of 4 mm, which is cooled to below 0 °C. The grey region indicates ice, the green region represents liquid water and the white region indicates air. (b) The real melting images recorded by confocal microscopy. In panel 1 the entire field of view is frozen (the bright region is due to the accumulation of fluorescent dye). In panel 2 melting occurs within 70 ms, replacing the ice with liquid water and a thin air gap with a thickness of 10 μm to 20 μm. In panels 3 and 4 the liquid water aggregates into circular micro-droplets. Also see Movie M1.†

We used deionized water dyed with rhodamine B (concentration = 0.7 mM) as the sample, and observed its freezing process with a confocal microscope (Leica SP5, objectives: 10× oil and 63× oil). Due to the thin depth of field (<10 μm) of confocal microscopy, we can visualize the freezing process right at the substrate–water interface very clearly, and obtain detailed information of the ice attachment onto the substrate. The water droplet is contained in a copper well (diameters: 1.5 mm, 3 mm, 4 mm and 5 mm) which is drilled through a copper plate (size = 12 cm × 4 cm, thickness = 1.4 mm) and cooled by a pair of Peltier plates (TEC2-25408T125, Xindahengye Electro, China) which were attached to the two ends of the copper plate with heat-conductive grease. A glass slide (thickness = 170 μm) was attached to the bottom of the well with epoxy (Versachem, 5 Minute Epoxy, thickness ≈ 100 μm) and served as the substrate (the detailed experimental set-up is presented in the ESI, Fig. 1†). With this experimental set-up, the water droplet can be cooled down to lower than −20 °C. We put multiple thermistors (Measurement Specialties, MCD Probe 10k RES) inside the droplet combined with a multimeter system (Keithley, Model 2700) to measure the temperature variation during the entire process. After the freezing was completed, we measured the adhesion force with a force sensor (Sandoo, SN200, resolution = 0.1 N) similar to previous studies.^30,31^

## 3 Results and discussion

Freezing of a super-cooled water droplet consists of two stages:^10,32^ a rapid first stage, during which a small fraction of water freezes and at the same time a temperature jump from the supercooled temperature to zero degrees occurs, and a much slower second stage during which the rest of water freezes and the temperature remains close to zero degrees. Here we focused on the second stage, because the sudden melting event occurred in this stage.

In the second stage, freezing starts from the wall of the copper well (diameter = 4 mm) and then propagates towards the center. During this process, ice forms and adheres to the bottom glass substrate.^33–35^ At the same time, lots of air previously dissolved in water is expelled out of the ice due to the phase transition, and produces many bubbles^36^ and channels inside the bulk ice. The channels can connect to each other and also connect to air bubbles, making interconnecting air networks. These networks are mostly filled with air, plus a little bit of unfrozen water flowing along the channels (see the Movie M1 in the ESI†). When the freezing is close to the end, the ice at the ice–substrate interface suddenly melts and forms a thin gap between the bottom substrate and the bulk ice. Melting also produces some small droplets with diameters between 1 μm and 40 μm, which we call micro-droplets, shown by the bright green spots in Fig. 1(b) and Movie M1.† The entire melting process is quite rapid and takes less than 0.1 s.

We schematically demonstrate the overall picture of the sudden melting event at the substrate–ice interface in Fig. 1(a): as the system is continuously being cooled, the freezing front moves from the copper wall towards the center, as shown by the first panel. Here we demonstrate the situation adjacent to the bottom substrate. The grey area indicates ice and the green region represents liquid water. However, before the icing front at the bottom substrate reaches the center and completes the freezing process, the ice previously formed suddenly melts, in the direction of the edge from towards the center, as illustrated in the second panel by the white area. This melting event makes the bulk ice there detach from the bottom substrate, and produces a gap around 10 μm to 20 μm thick, as represented by the white region. The newly appeared water from melting subsequently aggregates into micro-droplets, as indicated by the green spots. However, during and after the melting, the central liquid region continues to freeze, and the bulk ice only attaches to the bottom substrate at the central grey region (around 0.2 mm^2^) while the rest of the area is detached, as demonstrated in the third and the fourth panels. Such melting and detachment behavior significantly reduces the adhesion between bulk ice and the bottom substrate, and may help to achieve an effective anti-icing method.

We illustrated the sudden melting event by directly visualizing a small region in Fig. 1(b): with the fast resonant scanning of confocal microscopy, we could image the dynamics within a thin slice of the region right above the bottom substrate. In panel 1 of Fig. 1(b), the entire field of view was frozen, with a bright region caused by the accumulation of fluorescent dye previously dissolved in water but later expelled by ice during freezing. In the entire field of view there was essentially very little liquid water and the region was frozen almost completely. However, within 70 ms the frozen area suddenly melted, replacing the ice with liquid water and a thin gap of air (10–20 μm), as demonstrated in panel 2 (see also Movie M1† for a better illustration). Note that this melting event only occured in a narrow layer right above the substrate with a thickness of 10 μm to 20 μm, and most of the bulk ice was still intact. In panels 3 and 4, the liquid water aggregated into circular micro-droplets while the rest of the dark region was air, and the bulk ice detached from the bottom substrate throughout the field of view.

**Fig. 2:**
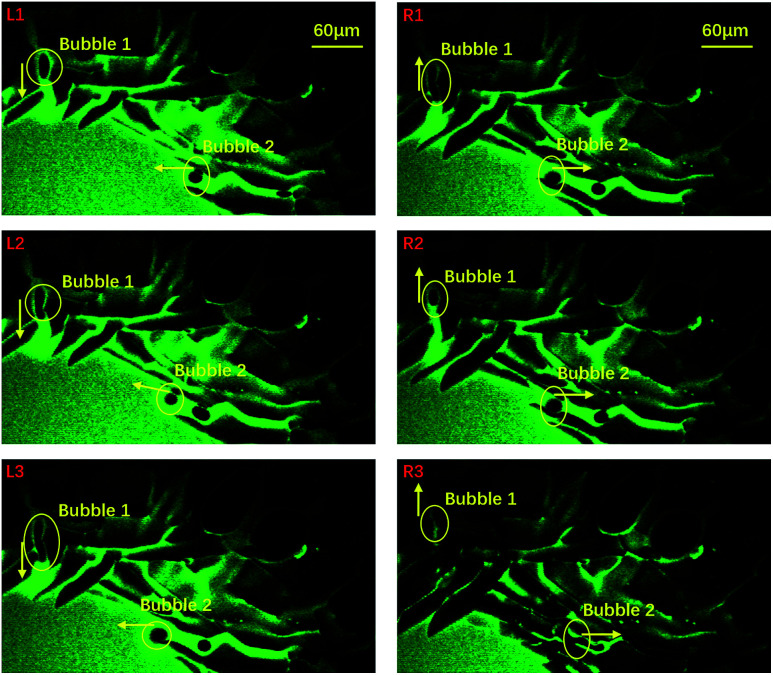
An indication of the time span covering the sudden melting event taken by 10× oil objective confocal microscopy. We selected 6 images which represent 6 typical positions during the evolution process. Image L1 to L3 indicate that the bubbles were moving to the central area while R1 to R3 indicate that the bubbles were moving in an opposite direction. The yellow arrows symbolize their motion direction and their ends stand for the right positions. The green and dark areas stand for water and ice, respectively. The corresponding movie is included in the ESI, M2.†

To probe the origin of the melting, we focused on the central area and studied the liquid flow by following some air bubbles at a frame rate of 30 frames per second. During the freezing process, many channels formed between neighbouring ice crystals and thus air bubbles and liquid water could flow in these channels, as shown in Fig. 2: the left three panels show that two air bubbles moved towards the center in the early stages of freezing. However, in the right three panels, we see that the two bubbles reversed their directions and traveled from the center towards the edge. Right after this flow reversed, the melting event occurred. Clearly, the sudden melting event is closely related to the flow of liquid water.

How can we understand the correlation between the water flow and melting? To address this question, we measured the temperature of liquid water in the central area, and it was around 1 °C approximately (see the ESI, Fig. 6†) and above the melting temperature. Therefore the liquid water at the center is warmer than the ice crystals on the substrate (usually −5 °C or below). Once this warm water reverses its flow direction and enters the channels at the bottom substrate, it can induce the sudden melting event observed before. Therefore, we argue that the counter-flow of warm water should be a trigger for melting.

To verify this, we performed experiments with water degassed at a pressure of 176 ± 4 Pa. Once degassed, there were no channels and air bubbles upon freezing, therefore no counter-flow could travel along the channels. As expected, the sudden melting event did not show up anymore, as shown in the ESI, Fig. 7.† However, after we put the degassed water under an ambient atmosphere for 2 hours and froze it again, the channels and air bubbles reappeared, as did the counter-flow and the sudden melting event. It is obvious that the reappearance of melting is due to the air dissolved in water.

We also performed freezing experiments on a sapphire substrate, which has a very high thermal conductivity. The sapphire substrate makes crystallization not only initiate from the copper edge, but also from the substrate itself because of its high thermal conductivity (27.2 W m^−1^ K^−1^ at 300 K). As a result, air bubbles and channels cannot form near the water–substrate interface like the ones previously formed on a glass surface.^37^ Therefore, no counter warm water flow and sudden melting can occur on the sapphire substrate.

**Fig. 3:**
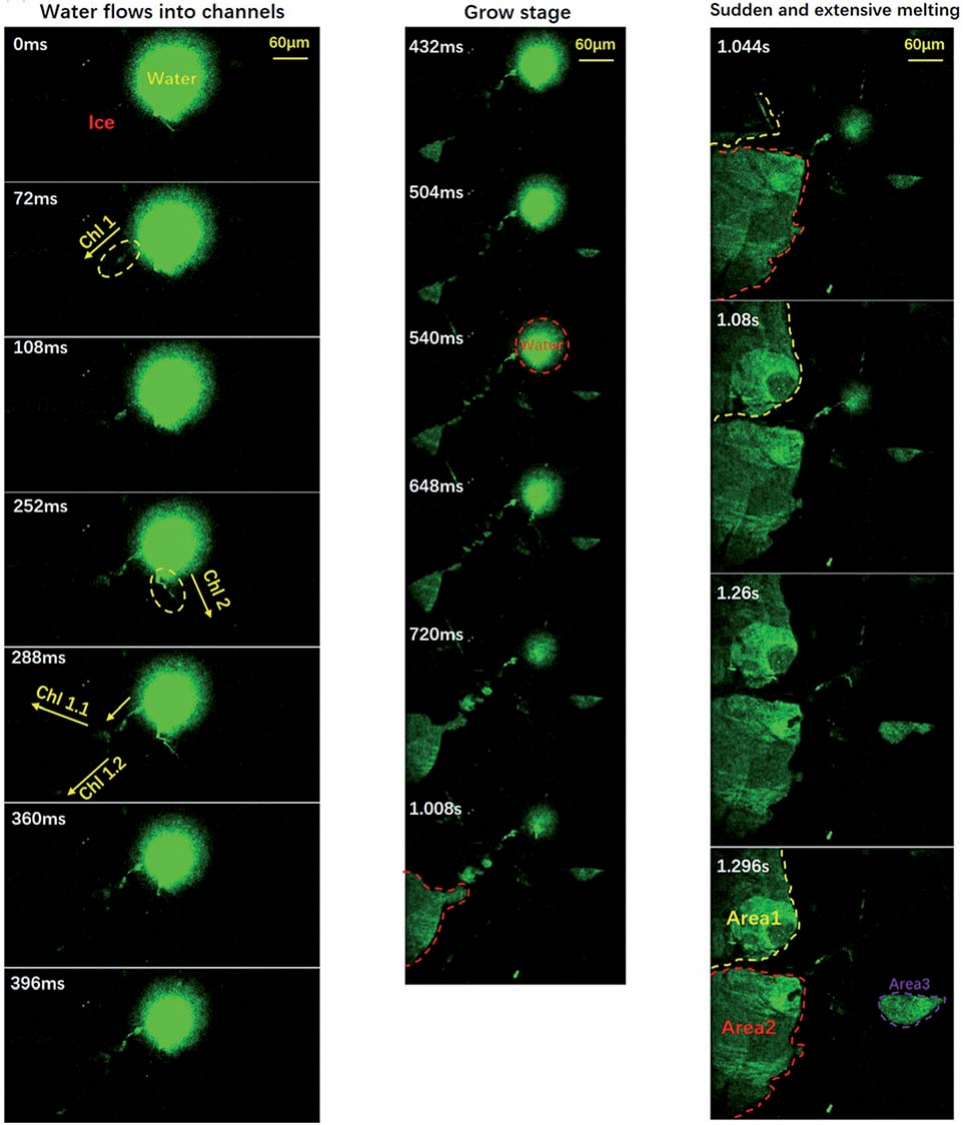
Visualization of the freezing process of water using a relative low gas content. The pressure is about 247 ± 4 Pa. The evolution of melting area during stage two is divided into three stages: the flowing stage, the growing stage and the sudden melting stage. Green areas indicate liquid water and the dark areas indicate ice and air. Time resolution = 36 ms. The movie is included in the ESI, M3.†

To clarify the detailed process of the melting event, we conducted experiments with partially-degassed water (at a degas pressure of 247 ± 4 Pa). Due to the small amount of air, we can illustrate the melting process in a cleaner manner, as shown in Fig. 3.

The entire process could be divided into three stages: the flowing stage, the growing stage and the sudden melting stage, as demonstrated by the left, middle and right panels. In the flowing stage, only a few sparse channels formed (three in this case). Because of the sparse channels, we could visualize the detailed process caused by each channel, which is clearer than the normal-water situation where many different channels interfere with each other. Note that the size of the central liquid water now was ≈0.01 mm^2^, only 1/20 of the size shown in Fig. 1. We can clearly see that liquid water flowed into three channels from the central liquid area. In the middle panels of the growing stage, liquid water kept flowing into the channels and three areas began to melt and grow. But, the growing speeds were quite low (area 1: ∼ 0.015 mm^2^ s^−1^, area 2: ∼0.036 mm^2^ s^−1^, and area 3: ∼0.02 mm^2^ s^−1^). Suddenly, as shown in the right panels, the melting areas expanded dramatically, within a short period of less than 100 ms. Note that area 1 and area 2 connected to each other. To sum up, the melting started slowly in some areas connecting to the central liquid region *via* channels, and suddenly expanded dramatically to trigger the sudden melting event. This is well consistent with the proposal in Alsayed’s paper in 2005.^38^ When the expansion areas intercepted, they combined to form a huge area of liquid which was green in most of the field of view. By this simplified and essential scenario, one can figure out that the melting mechanism can be concluded as ‘initiation from all of the channels and then expansion to the whole substrate’. This is due to the many channels from the air bubbles. With this well-reproducible visualization, clearly the dissolved air forms channels which enable the counter-flow of warm water, and the flow of warm water towards the edge triggers the sudden melting event.

**Fig. 4:**
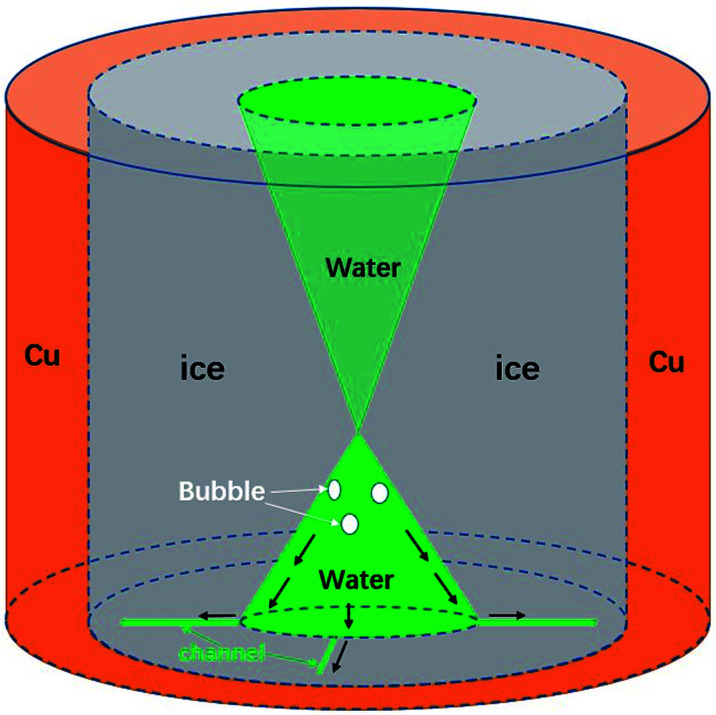
Schematic of the cone shape of the 3D structure of the sample droplet in stage two near the sudden melting event. Green areas indicate liquid water which is like an hourglass, closed in the middle area. Gray areas indicate ice crystals. White ellipses represent bubbles. The dark arrows indicate the flow directions of water when the counter-flow happens.

However, the underlying mechanism of the counter-flow is still unknown. By rapidly tuning the focal plane up and down (much faster than the freezing rate) during the freezing process, we can capture the overall shape of the icing front in 3D, as shown in Fig. 4. Clearly it had the shape of two cones connecting at the tip. This shape was due to the freezing speed being the fastest in the middle of the sample, causing an ice dome sealing some liquid water below.

Through a further comparison between the initiation time of the melting event and the formation of the ice dome, it was assured that the dome was 700 μm to 800 μm above the substrate surface, regardless of the cooling rate. The upper cone shape was also supported by Alvaro’s paper^39^ and other related publications.^40–42^ Combining the cone shape of the liquid water region and observations of the counter-flow, we believe that high liquid and air pressures are the cause of counter-flow. More specifically, a continuous production of air bubbles from freezing, together with the volume expansion of freezing, increases the liquid and air pressure under the ice dome.^43^ Eventually it reaches a critical value, which drives air and liquid water into channels and the flow outward. We also tested the influence of the size of the copper hole in the formation of counter-flow, as shown in the ESI, Table 2.† It turns out that the critical size of the central liquid water area almost remains as a constant (0.2 mm^2^), regardless of hole size and other factors.

In conclusion, air expelled by solidification produces channels between ice crystals, which enable air and water to flow along them. After the formation of the ice dome, air and liquid pressure under the dome increases, eventually reaching a critical value and driving warm water towards the edge along the channels. This warm water triggers the melting of the previously formed ice crystals.

**Fig. 5:**
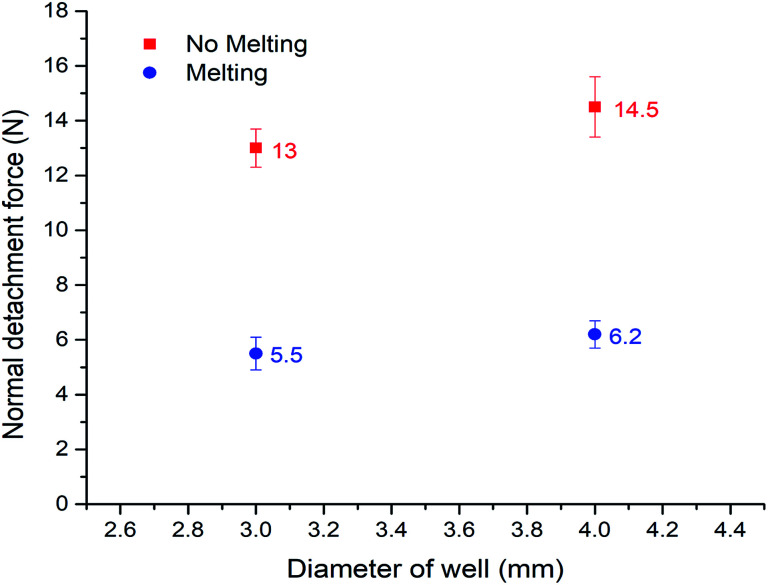
Measurement of ice adhesion forces for the two cases. Each data point here is an average of 10 data from 10 measurements under the same conditions. The error bar of each data point is also presented.

On the basis of such an amazing phenomenon, ice can detach from the bottom substrate after melting. Therefore it may become a highly promising anti-icing method which is novel and completely different from the prevalent method of fabricating super-hydrophobic surfaces. To quantitatively test this application, we constructed a set-up similar to Varanasi^30,44,45^ to evaluate the ice adhesion force. The result is shown in Fig. 5 (the related set-up is included in the ESI, Fig. 2†). We compared the adhesion of normal water that has the sudden melting process, with degassed water which does not have it, in two different copper well sizes (diameters = 3 mm and 4 mm). The ‘sudden melting event’ happened in the case of normal water but not in the degassed water situation. As expected, more than a 50% deduction of adhesion was verified (5.5/13 N and 6.2/14.5 N). The data shows clearly that our method is quite effective. Therefore, by carefully designed holes on the surface, we can achieve detachment of ice from the bottom substrate, suggesting an effective anti-icing method. Compared with the prevalent super-hydrophobic surface technique, our approach only requires millimeter-sized wells instead of complex microscopic textures. Therefore, it is much easier and cheaper to produce, as well as much more robust for large-scale practical applications.

## 4 Conclusion

In summary, through a lot of carefully designed experiments, we discovered a novel and stunning self-melting phenomenon in the second stage of freezing of water droplets which we call a ‘sudden melting event’. It happens only in a depth of 10 μm to 20 μm above the water–substrate interface, but throughout all the interface except the most central area, while the droplet keeps cooling down. After this event, the majority of the area of the ice–substrate interface is replaced by water and air. We also conducted careful experiments to find its mechanism. By degassing the sample to different degrees of vacuum, we found that the air (once dissolved in water) excluded by water freezing produced inter-connecting channels in the bulk ice, which transported the warm water produced by latent heat to the substrate and caused the sudden melting event. Since this event greatly decreases the contact area between ice and substrate, we designed a force sensor to measure its influence on the ice adhesion force. The data shows more than a 50% deduction which is extremely amazing. Because our approach only requires millimeter-sized wells and glass slides, it is quite easy and cheap to build. Therefore, we are convinced that it is very promising for large-scale practical applications.

## Conflicts of interest

There are no conflicts to declare.

## Supplementary Material

RA-008-C8RA06601A-s001

RA-008-C8RA06601A-s002

RA-008-C8RA06601A-s003

RA-008-C8RA06601A-s004
