# Supplementary material for: A sudden-melting event during water freezing inside a copper well
Source: RSC Adv. 2018 Oct 15;8(61):35257–62. doi: 10.1039/c8ra06601a (PMC9088081; doi:10.1039/c8ra06601a)
Supplement: RA-008-C8RA06601A-s004 [file RA-008-C8RA06601A-s004.pdf]

Electronic Supplementary Material (ESI) for RSC Advances.
This journal is © The Royal Society of Chemistry 2018

Supplementary Information

(a)

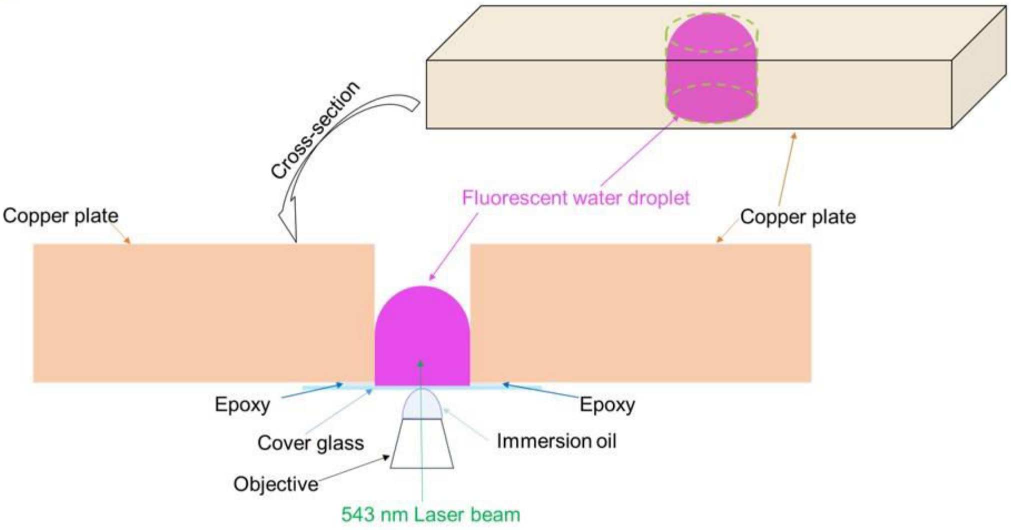

(b)

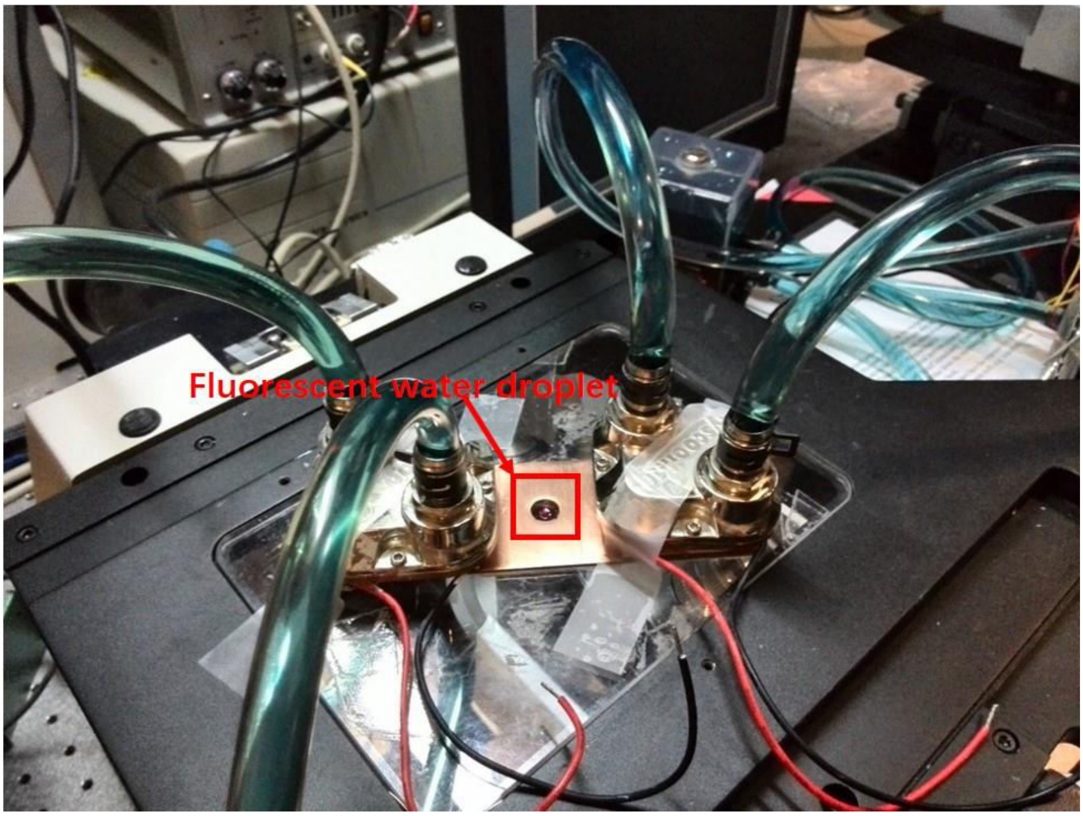

Fig.1: (a) The schematic figure of experimental set-up. The bottom section is the cross-area view, and upper section is the perspective view. Fluorescent water droplet is indicated by violet dome. Bronze bricks stand for copper plate. (b) The real image of our experimental set-up. Blue tubes indicate cooling system.

Figure 1 shows the schematic and real image of our self-made experimental set-up. We use the epoxy (VersaChem 5 Minute Epoxy) to attach glass substrate to copper surface for its low heat conductivity. And to obtain a stable and clear enough image, we prefer immersion oil objective instead of dry objective. The laser with a 543nm wavelength is selected as our incident laser based on the excitation spectrum of rhodamine b (λ_{max}=553nm_Sigma). When we need to conduct the deaeration experiment, we attach one more cover glass on top surface of copper hole with epoxy. And the Peltier plates are placed at both sides of copper plate. Under this configuration, nucleation initiates from the inner wall of copper well and then extends to glass surface.

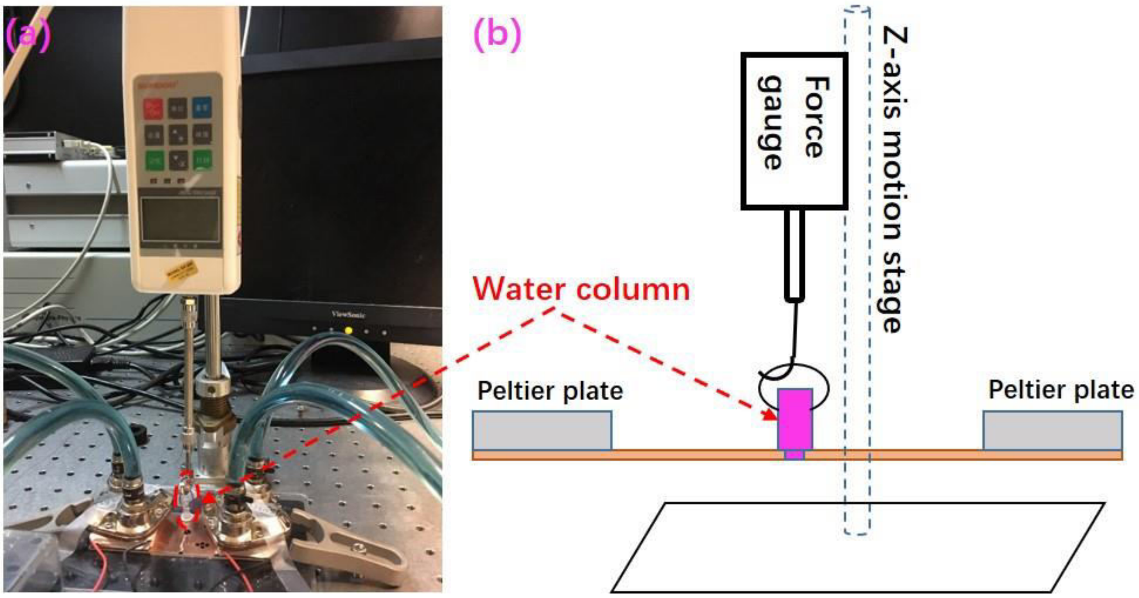

Fig.2: The self-made set-up of ice adhesion strength measurement. (a) The real set up. (b) The schematic illustration.

This figure shows our experimental set-up to measure ice adhesion force. The thickness of the copper well is 900um which is larger than the height of the tip of the ice dome. We use a force gauge (resolution=0.1N) which is placed in a vertical direction to measure normal force. And the gauge is connected to computer, thus we can monitor force variation in real time. Blue pipes indicate our cooling system. And we used a stainless-steel column to contain the water overflowing the mouth of the well. To minimize the interference of the adhesion between the column and copper surface which is from the iced vapor, we placed moderate plasticine surrounding the column but not sticking to the column.

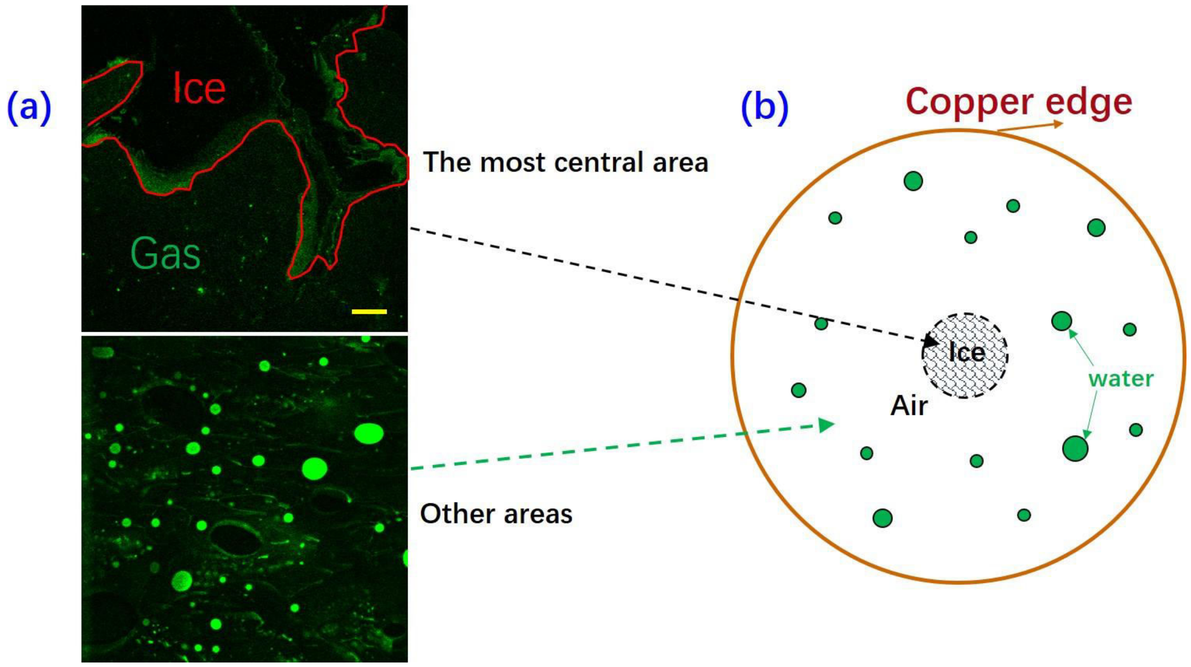

Fig.3: The configuration of substrate-water interface after sudden melting event. (a) Real images of central area (ice enclosed by a red curve) and micro droplets indicated by green dots. (b) Schematic of whole configuration. Scale bar= 40μm.

Figure 3 presents the configuration of the water-substrate interface after sudden melting event. It is evident that there are only air and micro-droplets left in the melting gap but remains a tiny proportion of ice in central area.

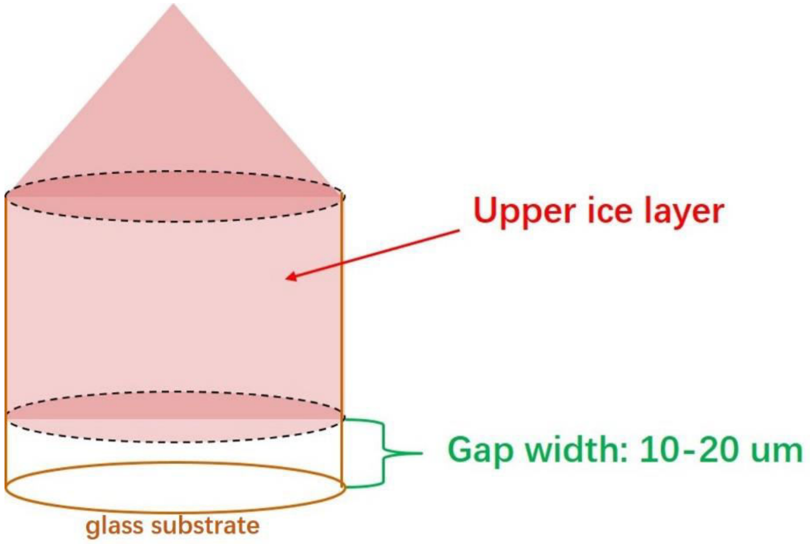

Fig.4: The schematic of the gap resulted from the sudden melting event in vertical direction. Bronze sections stand for bulk ice and the top tip. The gap is between bulk ice and substrate.

Since there are only micro-droplets and air in the melting gap (even though still with a tiny central ice), the gap can be regarded as a void which indicates an almost zero contact with the glass substrate. It further suggests a huge deduction of the ice adhesion.

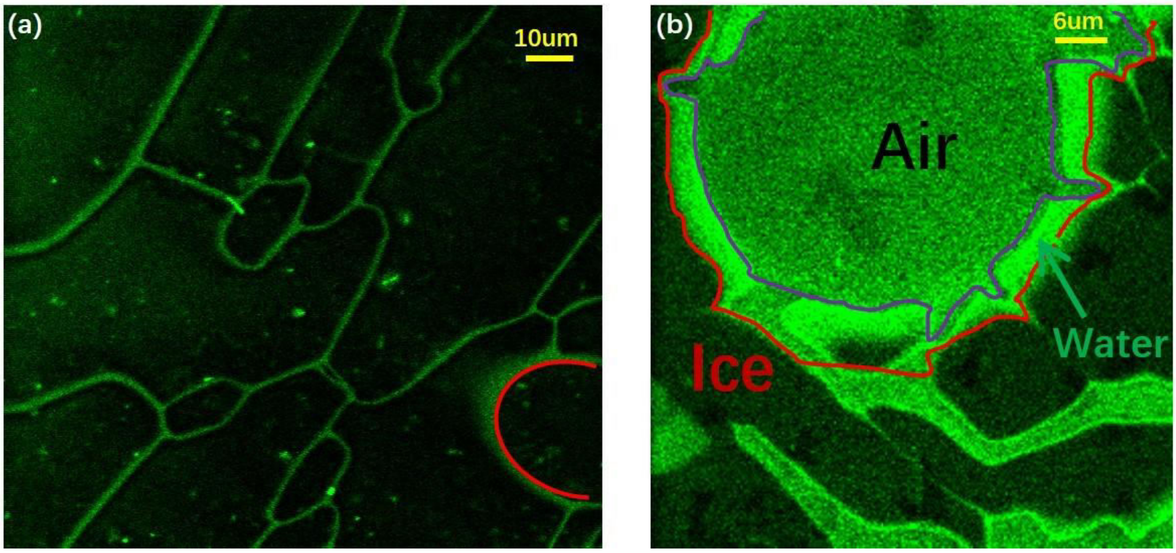

Fig.5: (a)The networked configuration of water-substrate interface in stage two of freezing process, under 10x magnification. There are lots of channels (bright green curves) connecting to each other and connecting to air cavities (marked with red curve). (b)The configuration of an air cavity, consisting of air, water and ice, under 63x magnification.

The configuration of water-substrate interface before sudden melting event is networked. We call it networked because it is occupied with lots of channels which connect to each other and connect to air cavities. The channels are gaps between ice crystals, as shown in part (a). And we used a 63x magnification objective to enlarge the details of an air cavity, as shown in part (b). An air cavity consists of air (filled in cavity), water (in-between air and cavity edge) and ice (surrounding cavities and channels). Channels connect to air cavities.

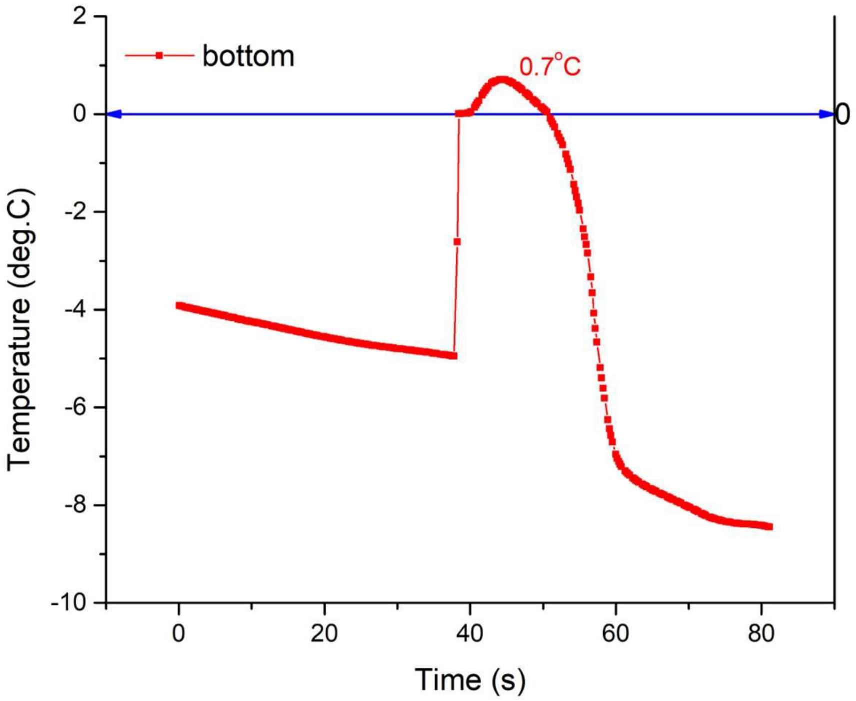

Fig.6: Temperature evolution of water-substrate interface during the whole freezing stage. The

temperature is acquired by a thermistor inserted just on substrate surface and positioned close to the central area. The steep increase near t=40s indicates the stage one. After that, stage two begins.

It is clear that the temperature of central water is larger than 0 degree centigrade (≈1℃). This temperature is much higher than ice temperature (usually -5℃ or below). When this part of water crystallizes, its temperature drops below zero degree centigrade.

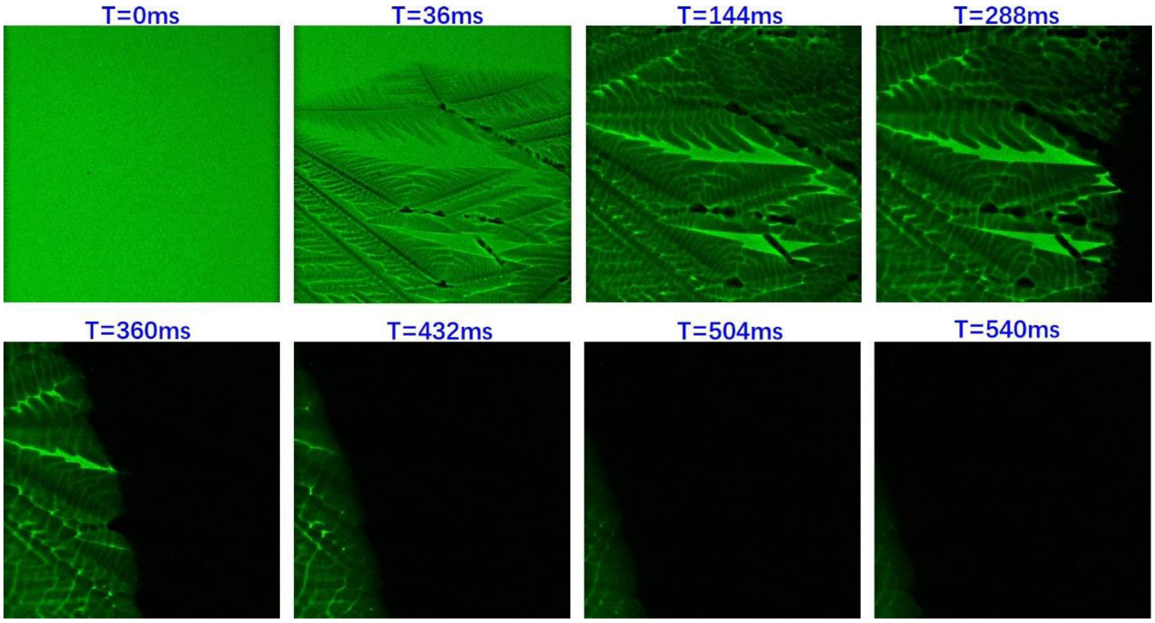

Fig.7: The freezing behavior of a degassed water sample (0.7mMol/L) on glass surface under 7V cooling voltage.

Under a degassed case (pressure=176±4Pa), the freezing behavior of this water sample shows no channels and air bubbles, only conspicuous dendritic ice crystals in stage one and dense ice crystals in stage two. And there is no sudden melting event subsequently. We keep other conditions the same with normal freezing case.

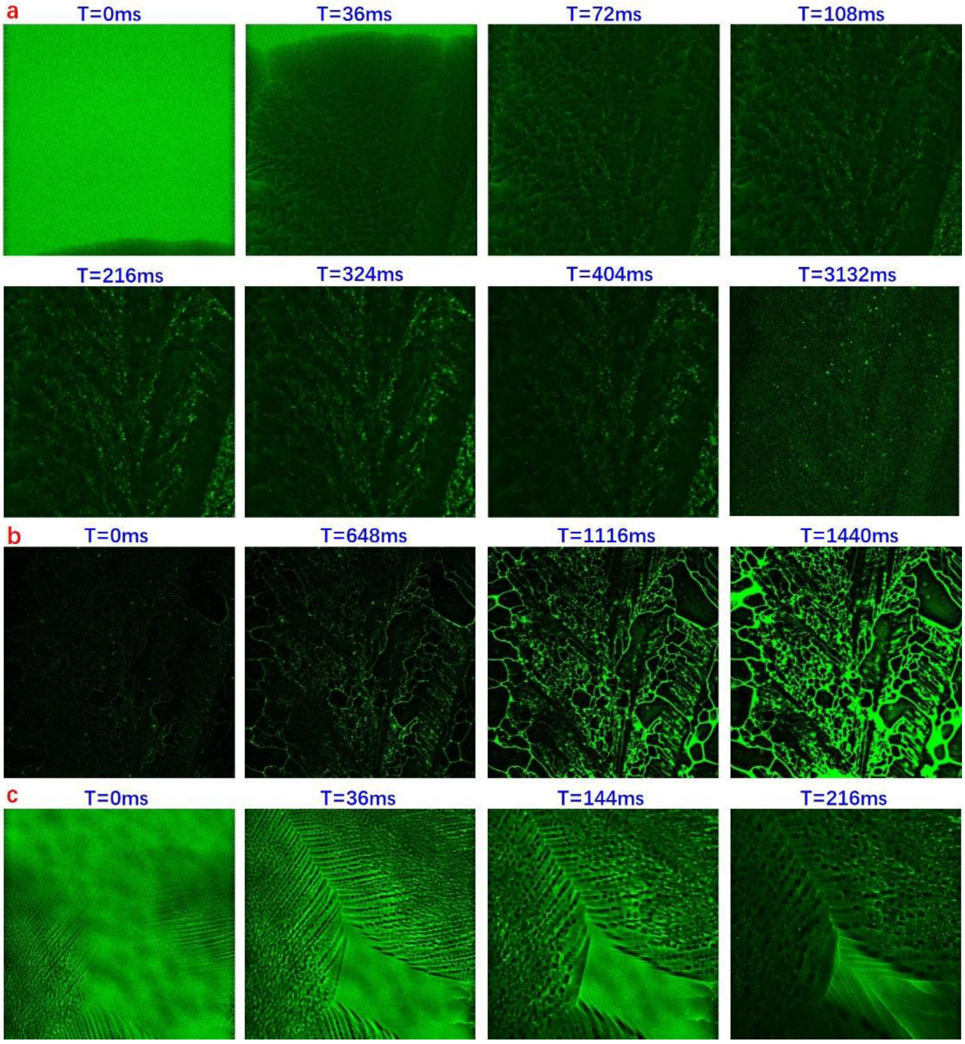

Fig.8: The freezing behavior of a normal water sample (0.7mMol/L) on sapphire substrate under 7V cooling voltage. (a) Direct observation of freezing process on water-substrate interface. (b) Corresponding melting while stop cooling. (c) Another freezing experiment similar to (a). Image size= 387.5μm x 387.5μm, Frame rate= 36ms.

Figure 8 shows a series experiments conducted with normal water and sapphire substrate. Sub-figure (a) and (c) represent the freezing process. One can directly notice that nucleation initiates from substrate not copper since it has several conspicuous nuclei. The gaps between ice crystals are almost invisible unless we stop cooling and let ice melt by warming up, shown in sub-figure (b). Evidently, there is no any channels and air bubbles and therefore no sudden melting event. Figure 7 and 8 imply a same point-when there is no air in freezing, then there are no channels and bubbles.

Material	Thermal Conductivity(w/m.K)
Water	$0.56(0^oC)$
Ice	$2.22(0^oC)$
Air	$0.024(0^oC)$
Glass	$1.05(0^oC)$
Epoxy	$0.35(0^oC)$

Table 1: The heat conductivity of several materials contained in experimental set-up. The data is obtained from wiki and all at zero degree centigrade.

Obviously, the heat conductivity of air is the lowest one and almost 1/30 of water and 1/100 of ice. Thus, it slows down the heat transferring.

We examined the relation between the central ice size and the diameter of the copper well, shown in Table 2.

	Exp1 (mm2)	Exp2 (mm2)	Exp3 (mm2)	Exp4 (mm2)	Exp5 (mm2)	Exp6 (mm2)	Average (mm2)	percentage
**D=5mm**	0.196	0.227	0.256	0.199	0.201	0.247	**0.221** ±0.026	**1.12%**±0.13%
**D=4mm**	0.202	0.214	0.241	0.266	0.235	0.222	**0.2315** ±0.022	**1.84%**±0.17%
**D=3mm**	0.149	0.152	0.159	0.161	0.157	0.164	**0.157** ±0.0056	**2.22%**±0.08%
**D=1.5mm**	0.221	0.249	0.213	0.209	0.245	0.237	**0.229** ±0.017	**12.9%**±0.96%

Table 2: The table of central ice area measured under different well diameter, ranging from 1.5mm to 5mm. Each diameter repeated for more than 5 experiments. The measurement uncertainty of each experiment is less than $0.01mm^2$.

The diameter range of copper hole is determined by two limits. The upper limit is a crystallization limit. If the well diameter is larger than 5mm, then droplet will not crystallize in our experimental set-up for the limited power of cooling system. And the lower limit is a capillary limit. If the hole diameter is less than 1.5mm, then water droplet cannot be injected into the well. From this table, we found that the central ice area is approximately equal to $0.2mm^2$ regardless of the hole size. Evidently, if we want to obtain minimal attachment of upper ice layer and meanwhile with a good enough cooling rate, the diameter of 3mm is our optimal choice.
